# CD19-Targeted Immunotherapies for Diffuse Large B-Cell Lymphoma

**DOI:** 10.3389/fimmu.2022.837457

**Published:** 2022-02-24

**Authors:** Massimiliano Gambella, Simona Carlomagno, Anna Maria Raiola, Livia Giannoni, Chiara Ghiggi, Chiara Setti, Chiara Giordano, Silvia Luchetti, Alberto Serio, Alessandra Bo, Michela Falco, Mariella Della Chiesa, Emanuele Angelucci, Simona Sivori

**Affiliations:** ^1^Ematologia e Terapie Cellulari, IRCCS Ospedale Policlinico San Martino, Genoa, Italy; ^2^Department of Experimental Medicine (DIMES), University of Genoa, Genoa, Italy; ^3^Laboratory of Clinical and Experimental Immunology, Integrated Department of Services and Laboratories, IRCCS Istituto Giannina Gaslini, Genoa, Italy

**Keywords:** monoclonal antibodies, antibody-drug conjugates, bispecific T cell engagers, genetic modification, engineered T cells, CAR-T cells, CAR-NK cells

## Abstract

Surgical resection, chemotherapy and radiotherapy were, for many years, the only available cancer treatments. Recently, the use of immune checkpoint inhibitors and adoptive cell therapies has emerged as promising alternative. These cancer immunotherapies are aimed to support or harness the patient’s immune system to recognize and destroy cancer cells. Preclinical and clinical studies, based on the use of T cells and more recently NK cells genetically modified with chimeric antigen receptors retargeting the adoptive cell therapy towards tumor cells, have already shown remarkable results. In this review, we outline the latest highlights and progress in immunotherapies for the treatment of Diffuse Large B-cell Lymphoma (DLBCL) patients, focusing on CD19-targeted immunotherapies. We also discuss current clinical trials and opportunities of using immunotherapies to treat DLBCL patients.

## Introduction

The 2016 World Health Organization Classification of Tumors defines Diffuse Large B-Cell Lymphoma (DLBCL) as a disease originating from mature B-cells, for a large proportion of which there are no clear and accepted classification criteria. Despite the DLBCL heterogeneity, the neoplastic cells typically express pan-B-cell markers CD19, CD20, CD22, CD79a, PAX5 (1), paving the way for the introduction of targeted therapies. Among these, the use of the anti-CD20 monoclonal antibody rituximab represented the cornerstone. Rituximab is a chimeric monoclonal antibody, whose murine variable regions bind to CD20 on B-cells, while the human constant regions mediate effector mechanisms (2, 3), such as complement-dependent cytotoxicity (CDC) and antibody-dependent cellular cytotoxicity (ADCC). Large randomized trials comparing standard chemotherapy alone to the addition of rituximab showed a clear survival advantage for the combined immunochemotherapy approach (4–6), leading to the association of chemotherapy to rituximab as the current standard of care for DLBCL patients (7). Despite the successful history of anti-CD20 immunotherapy in DLBCL, approximately 40-50% of patients ultimately do not respond to frontline treatment (8). Several mechanisms of resistance have been hypothesized, including CD20 loss, expression of CD20 variants lacking the determinants recognized by rituximab, and polymorphisms of FcγRIIIA negatively affecting effector cell functions (3), making the identification of alternative targets for immunotherapy a definitive need. Among pan-B-cell markers, CD19 is an attractive target due to both its broad presence through B-cell ontogeny and its functional role. CD19 is a 95 kDa, type I transmembrane glycoprotein whose expression starts early in B-cell maturation, concurrently with immunoglobulin gene D-J regions rearrangement in Pro-B cells, and ends with terminally differentiated plasma cells (9). The almost ubiquitous expression among B-cell lymphopoiesis of CD19 underlines its fundamental role in B-lineage functionality and commitment (9). In a murine model of B cell lymphoma, Chung and coworkers demonstrated a correlation between CD19 mRNA levels and the oncogene MYC expression, suggesting a role of CD19 in lymphomagenesis and arguing that CD19 ligation through targeted agents could represent a strategy to disrupt MYC signaling and interfere with oncogenesis (10). At present, four classes of drugs have been designed to target CD19: unconjugated monoclonal antibodies (mAb), antibody-drug conjugates (ADC) and molecules that specifically recruit T-cells, including bispecific T cell engagers (BiTE) and chimeric-antigen receptors (CAR).

## Monoclonal Antibodies

*Inebilizumab (MEDI-551)* is a humanized, a-fucosylated anti-CD19 antibody developed from the murine HB12b mAb through a two-step process: 1) HB12b humanization and Fab rearrangement, respectively to reduce immunogenicity and optimize CD19-affinity and 2) fucose removal to increase affinity for human CD16/FcγRIIIA, optimizing antibody-dependent cell cytotoxicity (ADCC) performed by Natural Killer (NK) cells and macrophages (11). Interestingly, subsequent observations underlined the role of F/V 158 FcγRIIIA polymorphisms in NK cell-mediated killing: in an *in-vitro* assay, heterozygosity for the high-affinity FcγRIIIA (namely, 158V allotype) was sufficient for efficient B-cell leukemia cells killing, while homozygosity for the weak-binding allotype FcγRIIIA (i.e. 158F allotype) was associated with the absence of activity (12). Despite a phase 1 trial showing both a good safety profile and some evidence of activity of single-agent inebilizumab treatment for B-cell malignancies, including DLBCL (NCT01957579), subsequent combination studies failed their endpoints, included a phase 1b/2 trial performed using inebilizumab in combination with an anti-PD1 mAb (NCT02271945).

*Tafasitamab (MOR208)* is an engineered antibody characterized by two amino acid substitutions, S239D/I332E enhancing FcγR and C1q binding and, therefore, effector cells recruitment (ADCC) and complement cascade activation (CDC) (13). In contrast to a-fucosylated antibodies, the S239D/I332E modification increases the affinity to all activating FcγR receptors (i.e. FcγRI, FcγRIIA, and FcγRIIIA) (14) irrespective of the FcγRIIIA-V/F allotype (15). A phase 2a trial with the single-agent tafasitamab showed promising activity in 35 patients affected by DLBCL (NCT01685008). Refractoriness to rituximab or FcγRIII-158F allotype did not impact tafasitamab treatment efficacy (16). Several combination trials, mainly with lenalidomide or bendamustine, are testing tafasitamab both in the relapsed and refractory (R/R) patients and first-line setting. The phase 2 L-MIND trial evaluated MOR208 plus lenalidomide for R/R DLBCL (NCT02399085), focusing on the synergic NK cell-mediated-ADCC observed when MOR208 is combined to lenalidomide (17), a potent NK cell activator (18). In this trial, 60% of the patients achieved a response and the median progression-free survival was 12.1 months (19). Moreover, an updated analysis showed activity even in high-risk categories (i.e. previously refractory) and beyond the second line (20). Based on L-MIND results, the tafasitamab-lenalidomide combination achieved the FDA approval for the R/R DLBCL. Currently, the phase 3 randomized, frontMIND trial (NCT04824092) aims to test tafasitamab plus lenalidomide in combination with the first-line chemotherapy regimen R-CHOP.

## Antibody-Drug Conjugates

*Denintuzumab mafodotin (SGN-CD19A)* is a humanized anti-CD19 monoclonal antibody conjugated with monomethyl auristatin F (MMAF), a synthetic analogue of the natural antimitotic agent dolastatin 10. As a tubulin-binding molecule, dolastatin exerts its cytotoxic effect through the inhibition of microtubule assembly and tubulin-dependent GTP hydrolysis, leading to cell cycle arrest and apoptosis (21). MMAF differs from another auristatin derivative, monomethyl auristatin E (MMAE), for a C-terminal modification which is aimed to limit membrane permeability and reduce bystander and off-target toxicity (22). A phase 1 study (NCT01786135) demonstrated the safety of SGN-CD19A in the clinical setting of R/R B-cell NHL, with 30% of evaluable patients achieving a complete response (23). Two subsequent studies with denintuzumab mafodotin in combination with chemotherapy were interrupted with no further development (NCT02592876; NCT02855359).

*Loncastuximab tesirine (ADCT-402):* upon ligation, CD19 is rapidly internalized, making it an ideal target for immune-conjugates, which carry highly cytotoxic molecules directly within the cell. ADCT-402 is composed of the humanized anti-CD19 antibody RB4v1.2 linked with tesirine (SG3249), a drug-linker which delivers, through lysosomal degradation, the pyrrolobenzodiazepine (PBD) dimer warhead SG3199. SG3199 forms a covalent bond with the minor groove of DNA (24) through a minimal distortion of the DNA helix, hence slowing DNA repair and promoting intracellular persistence (25). Moreover, once released by damaged CD19^+^ cells within the medium, its high permeability allows bystander cytotoxicity, even among CD19^–^ cells (26). Interestingly, loncastuximab might not preclude a subsequent CD19-targeted therapy: a small series of 14 patients who failed loncastuximab conserved CD19 expression and responded to anti-CD19 CAR-T cells (27). A phase 1 study (NCT02669017) in patients affected by R/R B-cell NHLs has shown a good safety profile and encouraging activity (28), confirmed by the phase-2 LOTIS-2 trial (NCT03589469), where single-agent loncastuximab achieved an overall response rate (ORR) of 48%, half of which in complete remission (CR) (29). Loncastuximab is being tested even in combination with targeted molecules such as ibrutinib, venetoclax or durvalumab (NCT03684694; NCT05053659; NCT03685344) in the R/R setting, as well as a first-line agent, together with chemotherapy (NCT04974996).

## Bispecific T Cell Engagers

*Blinatumomab (MT103):* Bispecific T cell engagers (BiTE) represent the attempt to engage T cells in a polyclonal fashion, thus overcoming limits of clonal-specific response. Blinatumomab is a bispecific antibody composed of four variable domains, oriented to form two single-chain antibodies (scFvs), respectively directed against CD19 and CD3 (30). A short amino acidic linker keeps the two scFvs together and allows sufficient flexibility for the crosslink (31). Pre-clinical data with blinatumomab highlighted that T cells, once recruited, are much more potent effectors than NK cells and monocytes/macrophages; moreover, both CD8^+^ and CD4^+^ T cells can exert cytotoxic functions, independently of CD28 co-ligation or IL-2 exposition/exposure (31). The phase 2 study in R/R DLBCL (NCT01741792) showed remarkable activity and suggested a refinement in its administration to avoid neurotoxicity (32), a complication already emerged during phase 1 (NCT00274742). The phase 2/3 trial (NCT02910063) evaluated blinatumomab as a second-salvage strategy through a dose-escalating approach, to avoid toxicities. Despite efficacy (ORR: 36%) in a highly unfavorable cohort, only 46% of patients completed the first cycle, mainly due to concomitant disease progression (33); the dose-escalating approach might have hampered efficacy in patients with a rapidly progressive disease. As a consolidation strategy after the rituximab-chemotherapy-based first-line, blinatumomab was remarkably able to convert positive minimal residual disease (MRD) to negativity (NCT03023878) (33).

*TNB-486 CD19/CD3*: cytokine releasing syndrome (CRS) and neurotoxicity can represent life-threatening complications of CAR-T cells and BiTE therapies, limiting their use especially in frail patients. TNB-486 is a fully human, CD19/CD3 bi-specific antibody specifically designed to reduce the cytokine release from activated CD3^+^ cells upon engagement. The molecule is constituted of a high-affinity anti-CD19 heavy chain and a low-affinity anti-CD3 light chain, the latter with low-activating potential. *In vitro* models have demonstrated that the cytokine secretion (i.e., IL-2, IFN-γ, IL-6, IL-10, and TNF) by CD3^+^ cells is minimal even at saturating doses for tumor lysis (34, 35). A phase 1 study (NCT04594642) is currently testing TNB-486 for R/R B-cell non-Hodgkin lymphoma in patients who have received 2 or more prior lines of therapy.

## CAR-CD19 Engineered T Cells

### Chimeric Antigen Receptor – T Cells

Several aspects impact CAR-T cells biology, generating differences in expansion, persistence, and toxicity. Current evidence about relevant biological variables will be analyzed, together with a final update on the commercially approved products for DLBCL.

#### Chimeric Construct

The extremities of an anti-CD19 CAR construct, *in extenso* the extracellular scFv CD19 binding-region FMC63 and the intracellular CD3ζ signaling tail, are “fixed components” in the majority of products. Differences involve the hinge and the costimulatory domain (CD) which, respectively, optimize antigen-reach and prevent early exhaustion upon antigen-ligation. The combination of CD8α-derived hinge & transmembrane (TM) region with the 41BB CD (8-8-41BB CAR, adopted for tisagenlecleucel) is common. Alternatives are a full CD28 sequence (28-28-28 CAR, adopted for axicabtagene) or a combination of IgG4, CD28, and 41BB (IgG4-28-41BB, adopted for lisocabtagene). The incorporation of CD28 drives to a pronounced expansion, a favorable effector:target ratio and a faster tumoricidal activity, counterbalanced by a prolonged persistence for 41BB (36). In this view, CAR-T dynamics might be driven by downstream metabolic pathways: CD28 signaling leads to anaerobic glycolysis, typical of effector T-cells, 41BB to mitochondrial fatty-acid oxidation, and central-memory differentiation (37). A higher pro-inflammatory cytokines release might increase complications in CD28-based products (38). Interestingly, a clinical trial testing a 28-28-41BB product showed rates of inflammatory and neurological complications superimposable to 28-28-28 CAR-Ts, suggesting that the hinge-TM region, rather than the costimulatory domain, might be involved in mediating CAR-T-associated toxicity (38).

#### Manufacturing Process

It is composed of mononuclear cells apheresis and manipulation into the final product. Despite apheresis cryopreservation allows major flexibility, concerns may rise about post-thaw viability. Panch et al. confirmed a reduction of viable T-cells 2 days after thawing; nevertheless, in the presence of a sufficient apheresis, anti-CD19 CAR-T generation was not hampered (39). With regards to the final product, measures can be taken to control the CAR-T subsets composition and ratios. Sommermeyer et al. demonstrated that naïve (TN) CD4 and central memory (TCM) CD8 CAR-T cells have, separately, high anti-CD19 activity. Thus, hypothesizing a synergism, with CD4 producing IL-2 that activate and expand CD8 cells, they demonstrated that a fixed 1:1 CAR-T ratio of CD62L^+^/CD45RO^−^ CD4 TN and CD62L^+^/CD45RO^+^ CD8 TCM has the strongest activity against CD19 tumors (40).

#### Lymphodepletion

The lymphodepleting therapy consists in a course of chemotherapy, administered shortly before the CAR-T infusion to create a favorable immunological environment. Indeed, lymphodepletion increases chemotactic factors (MCP-1) and homeostatic cytokines (IL-2, IL-7 and IL-15), promotes eradication of regulatory T-cells and myeloid-derived suppressor cells, and the induction of costimulatory molecules. A combination of fludarabine and cyclophosphamide is the most employed regimen, relying on early trials where the addition of fludarabine to cyclophosphamide improved CAR-T expansion and persistence (41–43). Moreover Hirayama et al. demonstrated an association between higher doses of cyclophosphamide and a favorable cytokine profile (defined as day 0 MCP-1 and peak IL-7 concentrations) (44).

### Commercially Available Anti-CD19 CAR-T Products

#### Tisagenlecleucel (CTL019)

Tisagenlecleucel represents the first-in-class, autologous anti-CD19 CAR-T against DLBCL. Its approval followed the results of the phase 2 trial JULIET (NCT02445248) in R/R DLBCL. The manufacturing process consists in the lentiviral transduction of unselected T-cells, cryopreserved after collection (45). The JULIET trial tested tisagenlecleucel in 93 patients affected by R/R DLBCL, ineligible for or progressed after hematopoietic stem-cell transplantation. Half of the infused patients achieved a response, 40% of which as a complete remission. Promisingly, 65% of treatment-sensitive patients conserve a response (46). A trial update (47) and real-life experiences (48) support original data. Several trials involve tisagenlecleucel, included primary CNS lymphoma (NCT04134117) and pediatric R/R B-cell non-Hodgkin lymphoma (NCT03610724). The randomized, phase 3 BELINDA trial (NCT03570892) failed its aim to test tisagenlecleucel earlier as a second-line strategy (49). A phase 3 trial (NCT04094311) is investigating out-of-specification tisagenlecleucel for commercial release.

#### Axicabtagene Ciloleucel (KTEX19)

Axicabtagene manufacturing relies on the manipulation of a fresh apheresis and a gamma-retroviral transduction (50). KTEX19 was approved following the phase 1/2 study ZUMA-1 (NCT02348216), which exhibited remarkable results in a cohort of heavily pre-treated patients: 82% achieved a response, 54% a complete remission. Interestingly, responses were not negatively impacted by high-risk variables such as high IPI score, bulky disease and refractoriness to the previous line. A recent update showed that one-third of patients still in response at 24 months no longer had circulating CAR-T cells, suggesting that responses are not dependent on CAR-T persistence over time. Two multicenter trials are testing axicabtagene for high-risk DLBCL in an earlier setting: the ZUMA-7 (NCT03391466) as a second line, and the ZUMA-12 (NCT03761056) as a frontline treatment, respectively. Recent data from the ZUMA-7 demonstrated axicabtagene superiority in terms of overall response and risk of progression/death, in a comparison with a standard second line treatment comprehensive of high-dose chemotherapy followed by autologous transplant (51).

#### Lisocabtagene Maraleucel (JCAR017)

JCAR017 is a fixed 1:1 ratio of CD4 and CD8 cells (40). The manufacturing process, through which CD4 and CD8 T cells are separately activated and transduced through a lentiviral vector, leads to an enrichment in less differentiated, predominantly memory T-cells (52). The phase 1 TRANSCEND trial (NCT02631044) demonstrated high clinical activity (Response Rate 73%, Complete Remission 53%) with a low incidence of moderate/severe CRS and neurological events. The trial allowed the recruitment of secondary CNS lymphoma: in this subgroup, lisocabtagene achieved a 50% remission rate without fatal neurological events (53). A pooled analysis from 3 clinical trials (NCT02631044; NCT03744676; NCT03483103) in the outpatient setting provided encouraging data, with 46% of patients not requiring hospitalization after infusion (54). The TRANSFORM trial, aimed to compare lisocabtagene with high-dose chemotherapy followed by autologous stem-cell transplantation in a second-line setting, demonstrated a significant improvement in the probability of remission and a prolongation in event-free survival, in patients with early relapse or refractory disease (NCT03575351). Despite the need for a longer follow-up, an improvement in overall survival seems to emerge (55).

## Towards CAR-NK Cells

In order to overcome the hurdle of manufacturing timelines and the poor fitness of autologous T cells, two factors that can affect the CAR-T therapy efficacy, ongoing clinical trials (NCT03666000, NCT03939026 and NCT04416984) are testing allogeneic CAR-T products. In particular, treatment with PBCAR0191, an anti-CD19 CAR-T product in which endogenous TCR is disrupted by gene editing to prevent GvHD, together with an intensified lymphodepletion, has shown clinical benefit in the majority of NHL patients, yielding high rates of overall and complete response with promising activity in both CD19 CAR naïve subjects and those who progressed following auto-CD19 CAR therapy (56, 57). Other ongoing studies are testing ALLO-501/ALLO-501A, alternative allogeneic anti-CD19 CAR-T products modified by gene editing to disrupt the T-cell receptor alpha constant gene and the CD52 gene, respectively to reduce the risk of GvHD and allow the use of anti-CD52 mAb to delay host T cell reconstitution and graft rejection, have provided encouraging results (58, 59).

However, CAR-NK cells represent a more appealing alternative strategy to reduce the disadvantages related to the production and use of anti-CD19 CAR-T cells.

CAR-NK cells can be prepared in advance to be rapidly available on demand and, most likely, less capable of inducing CRS and neurotoxicity. Notably, CAR-NK cells can kill tumor cells even in a CAR-independent manner by their native receptors (including NCRs, NKG2D, DNAM-1, and activating KIRs), counteracting tumor escape mechanism due to lack of CAR-targeted antigen. Clinical-grade CAR-NK cells can be manufactured on a large scale starting from multiple sources, including NK92 cell line, peripheral blood mononuclear cells (PBMCs), umbilical cord blood (UCB), and induced pluripotent stem cells (iPSCs) (60–65).

The use of NK92 cell line can be advantageous for its unlimited ability to expand *in vitro*, even after repeated freeze/thaw cycles, but disadvantageous for their lack of some relevant NK receptors (including CD16), its potential tumorigenicity risk, and its low *in vivo* proliferation due to the irradiation needed before the infusion in the patient (66).

Differently, PBMC-derived NK cells may represent a good source for CAR-NK cell production (63, 67). Indeed, upon CAR-transduction NK cells maintain the expression of the main native activating receptors (NCRs, NKG2D, DNAM-1, CD16), can be administered without irradiation and, in a large fraction, exhibit a mature phenotype with high cytotoxicity. Moreover, each CAR-NK product obtained from a single donor can be used for the treatment of more patients in HLA-mismatched conditions. Finally, the limited lifespan of CAR-NK cells in the circulation and the reduced risk for GvHD allow repeated CAR-NK cells administrations (68).

Similarly, CAR-NK cells can also be produced from UCB NK cells, but, the limited amount of NK cells derived from a single UCB unit and the lower anti-tumor cytotoxicity of UCB-NK cells, mainly related to their less mature phenotype, represent obstacles (69, 70).

Finally, iPSCs have recently become an attractive source of CAR-NK cells for their unlimited proliferative capacity (71, 72). Indeed, CAR-engineered iPSCs can be induced to differentiate *in vitro* into hematopoietic progenitor cells and then into CAR-NK cells (72). Notably, from a limited number of iPSCs it is possible to obtain a large number of CAR-modified NK cells, even characterized by a homogeneous phenotype (73). However, even in this case, iPSCs-derived NK cells are usually expressing an immature phenotype (i.e. low KIRs/CD16 and high NKG2A expression).

In recent years, there has been a rapid increase in clinical trials using CAR-NK cells and investigating their possible application as therapeutic approach against hematological malignancies, including DLBCL (**Table 1**). Phase 1 and 2 of the pioneering clinical trial NCT03056339 enrolling 11 patients with R/R CD19^+^ malignancies, of which 2 DLBCL patients, showed promising results (74) and indicated the feasibility of adopting CAR-NK therapy for patients with high-risk B cell lymphoma and leukemia. Indeed, no patient infused with anti-CD19 CAR-NK cells, manufactured by transducing UCB derived NK cells (64), had shown neurotoxicity events, CRS, and GvHD. Moreover, 8 out of 11 patients (73%) had a clinical response, and 7 out of 11 (63%) achieved a CR. The maximum tolerated dose was not reached even with the higher infusion of CAR-NK cells (10^7^ CAR-NK cells per kilogram of body weight) and CAR-NK cells were detectable at low level for up to 1 year after infusion.

**Table 1:** Anti-CD19 CAR-NK mediated active clinical trials including DLBCL patients.

Identifier	NK cell origin	Construct	Location	First Posted	Status
**NCT03056339**	CB-NK cells	CAR.CD19-CD28-zeta-2A-iCasp9-IL15	USA	2017	Active, not recruiting
**NCT04245722**	iPSC (FT596)	CAR.19-NKG2D-2B4-CD3ζ-IL15RFhnCD16	USA	2020	Recruiting
**NCT04555811**	iPSC (FT596)	CAR.19-NKG2D-2B4-CD3ζ-IL15RFhnCD16	USA	2020	Recruiting
**NCT04887012**	iPSC (CAR-NK019)	Full construct undeclared (CAR.CD19, IL15 and modified CD16)	China	2021	Recruiting
**NCT04796675**	CB-NK cells	Full construct undeclared (CAR.CD19 and IL15)	China	2021	Recruiting
**NCT05020678**	PB-NK cells (NKX019)	CAR.CD19-OX40-CD3ζ-mIL-15	USA/Australia	2021	Recruiting

Others active clinical trials (NCT04245722, NCT04555811, NCT04887012) are registered to investigate the use of CAR-NK targeting CD19 derived from manufacturing iPSCs, however detailed results are not yet available. First evidences on the use of an anti-CD19 iPSCs-derived CAR-NK product (FT596 by Fate Therapeutics) in preclinical studies and clinical trials (NCT04245722, NCT04555811) suggest safety and well tolerability of the product (75, 76). FT596 is a CAR-NK product derived from iPSCs engineered to express a non-cleavable CD16 and IL-15 receptor fusion to promote additional functional activation (71). Its safety in advanced lymphoma treatment is under investigation both as monotherapy and as combined therapy with obinutuzumab or rituximab. A case of a heavily pre-treated DLBCL patient was enrolled in the first dose cohort of the study (lower infusion of CAR-NK cells - 30x10^7^cells) (77). A partial response has been observed upon infusion of one dose of FT596 that got better after a second infusion as proved by further decrease of tumor size and metabolism. The positive response to treatment wasn’t compromised by dose related toxicities and severe adverse effects, events of any grade of CRS, immune effector cell-associated neurotoxicity syndrome (ICANS), or GvHD (77) (https://ir.fatetherapeutics.com/news-releases/news-release-details/fate-therapeutics-reports-fourth-quarter-2020-financial-results).

Only a few months ago, the first clinical trial targeting CD19^+^ R/R B cell malignancies using CAR-NK cells obtained by engineering peripheral blood NK cells from healthy donors has been approved (NCT05020678). The purpose of this phase 1 study is to identify the optimal treatment dose with NKX019 product of Nkarta Therapeutics (https://ash.confex.com/ash/2021/webprogram/Paper146602.html). NKX019 expresses a CD19-targeted CAR, OX40 costimulatory domain, CD3ζ signaling moiety, and a membrane-bound form of IL-15 (mbIL-15) (78). Equipping CAR-NK cells with on-board cytokines, such as IL-15, lays the foundations for new therapeutic options aimed at improving clinical efficacy by enhancing both persistence and cytotoxicity against tumor cells (79).

## Discussion

**Figure 1:**
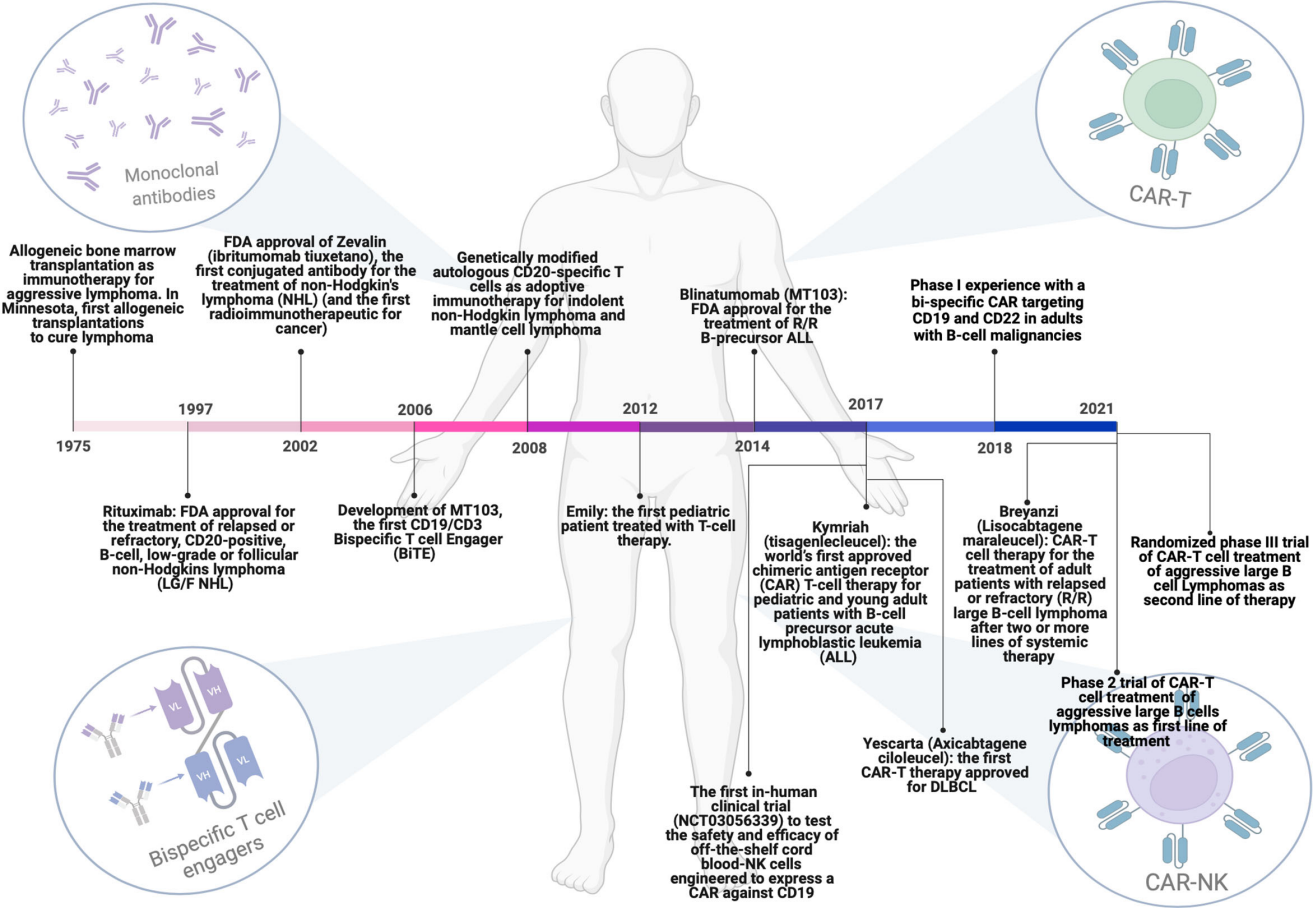
Milestones achieved over the years regarding the evolution of immunotherapeutic strategies for the treatment of DLBCL patients. From allogenic bone marrow transplantation to the use of monoclonal antibodies, Bi-specific T-cell engagers (BiTEs) and T or NK cells engineered with chimeric antigen receptors (CARs). This figure has been created using BioRender.

The optimization of mAbs production and cell therapies development have shown remarkable results and changed the clinical history of many tumor patients, even affected by DLBCL (**Figure 1**). The identification of stably expressed tumor-associated antigens to be targeted by immunotherapies and the improvement of the CAR structure are relevant issues to be explored in the next future. In this regard, simultaneous dual antigen targeting by tandem CARs could represent a way to overcome antigen loss by tumor cells and the subsequent, antigen escape-mediated relapse. The first clinical trial (NCT03097770) designed to evaluate the effect of an autologous, bispecific anti-CD19/anti-CD20 CAR-T in R/R B-cell lymphoma has shown its safety and ability to induce a durable antitumor response, possibly due to a superior immune-synapsis stability and the mitigation of antigen-negative escape by tumor cells (80).

Contemporarily with the improvement of anti-tumor efficacy, there is an urgent need to reduce the risk of significant, potentially life-threatening consequences of CRS and ICANS, which currently affect available CAR-T therapies. In this context, it has been demonstrated that activated monocytes and macrophages are the major source of IL-1 and IL-6 production during CRS and play a key role in the amplification of the inflammatory response (81). Currently, there is an effort to elaborate strategies aimed to target pro-inflammatory cytokines and their pathways contemporarily with CAR-T infusion with a prophylactic or pre-emptive purpose (NCT04432506, NCT04359784, NCT04148430) (82).

Furthermore, the choice of the adoptive immune cells to be modified with CAR is a critical field of investigation. In this context, NK cells represent an attractive source for genetically modified cellular immunotherapies (69, 83, 84). Unlike T cells, allogeneic NK cell infusions have reduced risks for GvHD and can be used to produce “off-the-shelf” products eliminating the need for a personalized product that is necessary for T cell-based therapies. Moreover, therapeutic approaches combining cell therapies with drugs, such as immune checkpoint inhibitors or ADCC triggering immunotherapies, could be exploited in order to target multiple tumor-associated antigens (85, 86) and further improve clinical outcomes.

In conclusion, we have many tools at our disposal, and others will certainly be developed in the coming years, that we can combine to further improve the clinical outcomes of patients affected by aggressive and still lethal cancers.

## Author Contributions

MG, SC, EA and SS designed and wrote the manuscript. CS designed and prepared the figure. LG, CGh, CGi, SL, AS, AB were involved in the search of the literature. AMR, MF and MD were involved in the revision of the manuscript. All authors listed have made a substantial, direct and intellectual contribution to the work, and approved the submitted version.

## Funding

Supported by the following grants: Fondazione AIRC IG 2017 Project Code 20312 (SS); PRIN 2017WC8499_004 (SS).

## Conflict of Interest

The authors declare that the research was conducted in the absence of any commercial or financial relationships that could be construed as a potential conflict of interest.

## Publisher’s Note

All claims expressed in this article are solely those of the authors and do not necessarily represent those of their affiliated organizations, or those of the publisher, the editors and the reviewers. Any product that may be evaluated in this article, or claim that may be made by its manufacturer, is not guaranteed or endorsed by the publisher.
